# Comment on "Hot pack therapy versus cherry seed pillow in fibromyalgia patients: a randomized controlled trial"

**DOI:** 10.1590/1806-9282.20251921

**Published:** 2026-08-21

**Authors:** Selin Cilli Hayıroğlu

**Affiliations:** 1 Istanbul Goztepe Prof. Dr. Suleyman Yalcin City Hospital, Department of Rheumatology – Istanbul, Turkey.

Dear Editor,

I read with great interest the article entitled "Hot pack therapy versus cherry seed pillow in fibromyalgia patients: a prospective randomized controlled trial"^1^. The study addresses a highly prevalent condition and evaluates a non-pharmacological, culturally familiar heat modality that is frequently used in daily practice. Given the growing interest in complementary and integrative approaches to chronic pain, examining such a low-cost intervention within a randomized controlled trial is timely and clinically relevant. At the same time, some key methodological and interpretative aspects merit closer scrutiny, as they have important implications for how clinicians should interpret the comparative effectiveness of cherry seed pillows versus standard hot packs.

The prospective randomized controlled design with computer-generated allocation and concealment is appropriate and helps minimize selection bias^1^. The use of a priori sample size calculation and validated outcome measures (Visual Analog Scale and Fibromyalgia Impact Questionnaire) is also commendable^2,3^. These design elements provide a solid framework for evaluating the comparative effects of the two heat modalities within a multimodal physiotherapy program^1,4^. Another important contribution of the research is its focus on a culturally accessible and safe home-based intervention. Cherry seed pillows are a common, low-cost household tool in Turkey, widely used for relieving muscle pain. By systematically evaluating their efficacy, the study bridges the gap between folk practices and evidence-based medicine. This perspective aligns with current trends in integrative health care, which aim to validate traditional methods through controlled empirical research.

However, several key methodological and interpretative aspects merit careful consideration.

First, although the authors conclude that cherry seed pillows are less effective than hot packs, this interpretation appears stronger than what the data conclusively support. Both groups received an intensive multimodal physiotherapy program (Transcutaneous Electrical Nerve Stimulation, ultrasound, and home-based exercise) 5 days per week for 4 weeks. This evidence-based background treatment is known to substantially improve pain and function in fibromyalgia, making the incremental effect attributable solely to the type of superficial heat relatively small^5^. In such a context, the trial is likely underpowered to detect modest between-group differences beyond the large within-group changes observed in both arms.

Second, the statistical emphasis is predominantly on within-group pre–post comparisons. For a randomized trial designed to compare two active interventions, the central inferential question is the between-group difference in change over time. An analysis of covariance or mixed-effects model adjusting for baseline scores would provide effect estimates and confidence intervals for this comparison. Without predefined minimal clinically important differences (MCIDs) and explicit between-group effect sizes, it is difficult to judge whether the numerically greater improvements in the hot-pack group are clinically meaningful or fall within a margin compatible with equivalence or non-inferiority.

Third, both interventions yielded very large within-group effect sizes for pain and FIQ, indicating that cherry seed pillows are not inert comparators but part of an effective, multimodal regimen^4^. From a clinical and public health perspective—especially in settings where access to formal physiotherapy or standard hot packs is limited—this aspect deserves stronger emphasis. A more cautious conclusion might therefore be that cherry seed pillows represent a potentially useful, though possibly slightly less potent, home-based heat modality rather than an ineffective alternative.

Finally, expectation and sensory differences between interventions may have influenced subjective pain reports. While assessor blinding was appropriate, participants could not be blinded to the type of heat used. Given the well-documented role of expectations in fibromyalgia pain perception, incorporating measures of treatment credibility and patient preference, or using sham or standardized-appearance devices, could help disentangle true physiological differences from contextual effects in future studies^5^.

In light of these considerations, the trial by Akpinar et al. still offers useful information for clinicians: both heat modalities, when integrated into a structured physiotherapy program, are associated with substantial improvements in pain and fibromyalgia impact^1^. However, we believe that a more nuanced interpretation of the comparative results—focusing on between-group effects, MCIDs and contextual factors—would provide a clearer message to readers regarding the true incremental benefit of standard hot packs over cherry seed pillows.

We commend the authors for addressing a commonly used, low-cost intervention in a rigorous design and hope that future studies will build on their work by incorporating prespecified MCIDs, between-group focused analyses, expectation measures and longer-term follow-up. Such refinements could help determine whether cherry seed pillows can be considered clinically non-inferior to standard hot packs in certain settings, and how best to integrate them into multimodal, patient-centred fibromyalgia care.

## Data Availability

The datasets generated and/or analyzed during the current study are available from the corresponding author upon reasonable request.
